# Pharmaco-EEG of antipsychotics’ response: a systematic review

**DOI:** 10.1192/j.eurpsy.2024.1391

**Published:** 2024-08-27

**Authors:** M. De Pieri, V. Rochas, M. Sabe, C. Michel, S. Kaiser

**Affiliations:** ^1^ HUG; ^2^University of Geneva, Geneva, Switzerland

## Abstract

**Introduction:**

Response to antipsychotic medications (AP) is subjected to a wide and unpredictable variability and efforts were directed to discover predictive biomarkers to personalize treatment. Electroencephalography abnormalities in subjects with schizophrenia were reported, as well as a pattern of EEG changes induced by APs

**Objectives:**

The aim of this review is to provide a synthesis of the EEG features that are related to APs efficacy, including both pre-treatment signatures and changes induced by APs during treatment.

**Methods:**

A systematic review of English articles using PubMed, PsychINFO and the Cochrane database of systematic reviews was undertaken in april 2023. Additional studies were added by hand-search. Studies having as an endpoint the relationship between AP-related clinical improvement and electroencephalographic features were included. Heterogeneity prevented a quantitative synthesis.

**Results:**

Out of 1232 records screened, 22 studies were included in a final qualitative synthesis. Included studies evaluated resting-state and task-related power spectra, functional connectivity, microstates and epileptic abnormalities. At pre-treatment EEG, the most relevant predictors of a poor response were a change in theta power compared to healthy control, a high alpha power and connectivity, a diminished beta power in resting-state. Considering EEG during treatment, an increased theta power, a reduced beta-band activity, an increased alpha activity, a decreased coherence in theta, alpha and beta-band were related to a favorable outcome.

**Conclusions:**

EEG is promising as a method to create a predictive biomarker for response to APs; further investigations are warranted to harmonize and generalize the contradictory results of reviewed studies.

**Disclosure of Interest:**

None Declared

